# Short-term exercise affects cardiac function ex vivo partially via changes in calcium channel levels, without influencing hypoxia sensitivity

**DOI:** 10.1007/s13105-021-00830-z

**Published:** 2021-08-27

**Authors:** Tytti-Maria Uurasmaa, Tomi Streng, Milla Alkio, Ilkka Heinonen, Katja Anttila

**Affiliations:** 1 Department of Biology, University of Turku, 20014 Turku, Finland; 2 Poznan University of Medical Sciences, Poznań, Poland; 3 Turku PET Centre, University of Turku, and Turku University Hospital, 20014 Turku, Finland; 4 Rydberg Laboratory of Applied Sciences, Department of Environmental- and Biosciences, University of Halmstad, Halmstad, Sweden

**Keywords:** Heart function, Ex vivo, Langendorff, Training, Pressure generation, CACNA, RyR, SERCA, NCX

## Abstract

Exercise is known to improve cardiac recovery following coronary occlusion. However, whether short-term exercise can improve cardiac function and hypoxia tolerance ex vivo independent of reperfusion injury and the possible role of calcium channels in improved hypoxia tolerance remains unknown. Therefore, in the current study, heart function was measured ex vivo using the Langendorff method at different oxygen levels after a 4-week voluntary wheel-running regimen in trained and untrained male mice (C57Bl/6NCrl). The levels of cardiac Ca^2+^-channels: L-type Ca^2+^-channel (CACNA1C), ryanodine receptor (RyR-2), sarco(endo)plasmic reticulum Ca^2+^-ATPase (SERCA2), and sodium-calcium exchanger were measured using western blot. Trained mice displayed lower cardiac afterload pressure generation capacity (rate and amplitude), but unaltered hypoxia tolerance when compared to untrained mice with similar heart rates. The level of CACNA1C positively correlated with the pressure generation rate and amplitude. Furthermore, the CACNA1C-RYR-2 ratio also positively correlated with the pressure generation rate. While the 4-week training period was not enough to alter the intrinsic cardiac hypoxia tolerance, interestingly it decreased pressure generation capacity and slowed pressure decreasing capacity in the mouse hearts ex vivo. This reduction in pressure generation rate could be linked to the level of channel proteins in sarcolemmal Ca^2+^-cycling in trained mice. However, the Ca^2+-^channel levels did not differ significantly between the groups, and thus, the level of calcium channels cannot fully explain all the functional alterations, despite the detected correlations. Therefore, additional studies are warranted to reveal further mechanisms that contribute to the reduced intrinsic capacity for pressure production in trained mouse hearts.

**Supplementary Information:**

The online version contains supplementary material available at 10.1007/s13105-021-00830-z.

## Introduction

Exercise is well known to benefit cardiac health and function. There is also strong evidence indicating that exercise decreases the risk of many diseases, such as coronary artery disease [12]. Coronary artery disease leads to decreased cardiac coronary flow and can lead to the myocardium becoming hypoxic and even ischemic if the coronary arteries are occluded. Owing to this, research focusing on ways to improve either cardiac coronary flow or hypoxia tolerance is of considerable importance.

It has previously been shown that exercise can improve cardiac function ex vivo by increasing left ventricular systolic pressure and the instantaneous first derivation of left ventricular pressure [31]. Cutilletta et al. (1979) were amongst the first to demonstrate that endurance exercise can increase the hypoxia tolerance of rat hearts in vivo [6]. According to their study, 8 weeks of treadmill exercise resulted in alterations in nervous system tone and the level of different circulating factors, which worked together to improve hypoxia tolerance. Exercise might also increase intrinsic cardiac hypoxia tolerance without neuronal regulation; however, this is an aspect that has remained relatively unexplored, with previous publications reporting contradictory findings. Wei et al. (1989), for example, demonstrated that a similar 8-week treadmill exercise regimen improved cardiac hypoxia tolerance in vitro [25]. However, improvements in heart hypoxia tolerance in vitro may not translate to significant improvements in vivo or ex vivo. In contrast, Fuller and Nutter (1981) found that a longer 12-week treadmill exercise regimen did not have a significant effect on isolated whole heart hypoxia tolerance in rats [8]. Their study focused on heart recovery from hypoxia and heart function during a brief period of hypoxia at one oxygen level. It is therefore possible that heart function and the effects of exercise on hypoxia tolerance would have differed at different oxygen levels. Since then, several studies have shown that long-term endurance exercise can increase the ischemic tolerance of ex vivo isolated rat hearts [4, 30, 31]. Even a short-term exercise period containing 1 week of weighted swimming has been shown to reduce infarction size [4]. In ischemic studies, the coronary flow is occluded for a set duration and the recovery of the heart is monitored after reperfusion. Therefore, these studies measure both hypoxia tolerance and tolerance to reperfusion injury. To our knowledge, it has not previously been studied whether short-term exercise can improve hypoxia tolerance of the heart independent of reperfusion injury by using an experimental design that investigates heart function ex vivo in multiple levels of hypoxia.

Other than hypoxia tolerance, endurance exercise is known to improve cardiac function. For example, a 4-week swimming exercise regimen causes a reduction in the resting heart rate of mice, a facet which has been shown to persist ex vivo and is the result of alterations in the funny channels HCN4 [7]. Additionally, exercise has been shown to alter the expression of other cardiac ion channels such as sarco(endo)plasmic reticulum Ca^2+^-ATPase (SERCA2) and sodium-calcium exchanger (NCX) [29]. Calcium channels such as L-type voltage-gated calcium channel (CACNA1C), ryanodine receptor (RyR), SERCA2, and NCX contribute to the cardiac excitation-contraction coupling by altering the calcium currents and concentration inside the cardiac cells. Calcium currents partly affect the speed of contraction [9, 32] while intra-sarcolemmal calcium concentration can directly affect the strength of the contraction by increasing the number of sites at which myosin and actin can interact [26]. However, few studies have focused on all four channels when looking at the effects of exercise on intrinsic cardiac function in mice.

Since most studies that investigate changes in heart hypoxia tolerance or calcium signaling have focused on long term exercise effects lasting 6–12 weeks [5, 8, 25, 29], there is a need to further investigate the effects of short-term exercise (4 weeks) on hypoxia tolerance and heart function, especially in mice which are used as in vivo models for numerous cardiovascular diseases. The small number of studies focusing on short-term exercise have indicated that 4 weeks of forced exercise is enough to induce resting bradycardia in mice and that 4 weeks of voluntary running wheel exercise increases the ventricular expression of brain natriuretic peptide and atrial natriuretic factor in mice [1, 7].

The aim of this study was to determine whether 4 weeks of voluntary wheel running exercise could improve cardiac function and hypoxia tolerance of the whole heart ex vivo and in multiple levels of hypoxia. The results of this study enhance our understanding of whether exercise can help the cardiac muscle to withstand hypoxia, a state which can arise in the vasculature as a result of, for example, coronary thrombosis. Additionally, the aim of this study was to determine the role of calcium channels, which contribute to cardiac contraction strength and velocity, in the possible functional alterations caused by short-term exercise. To achieve this, heart function was recorded continuously in decreasing oxygen levels using the Langendorff method, and western blot analysis was used to determine the levels of the different calcium channels. The hypothesis was that running exercise would improve the intrinsic hypoxia tolerance of mouse hearts and improve their function in normoxia and that these changes could at least partially be explained by alterations in the amount of calcium channels.

## Materials and methods

### Chemicals

Basic Tris (Sigma-Aldrich), bicinchoninic acid (BCA) assay (Pierce®, Thermo Scientific, USA), chloral hydrate (JT. Baker®, Deventer, Holland), cumene hydroperoxide (Sigma C-0524), DTNB (0.17 mM), formaldehyde (10 % Formalin, Sigma), heparin (LEO Pharma Oy, Finland), homogenization solution (62.5 mM Tris-HCl, 1μg ml^-1^ leupeptin, 1μg ml^-1^ pepstatin, and 1 mM PMSF, pH 6.8), H_2_O_2_ (200 mM, Baker), Krebs-Henseleit solution (119 mM NaCl 25 mM NaHCO_3_, 11.1 mM glucose, 4.7 mM KCl, 1.2 mM KH_2_PO4, 1.2 mM MgSO_4_•7H_2_O, 1.8 mM CaCl_2_•2H_2_O, oxygenated with 95% O_2_ 5% CO_2_), KF-Buffer (100 mM K-phosphate + 150 mM KCl solution, pH7.4), Laemmli solution (625 mM Tris pH 6.8, 20% glycerol, 2% SDS, 0.025% bromophenol blue, β-mercaptoethanol 5%), methanol (Merck), Mouse IgG RyR-2 antibody (Sigma-Aldrich R128), NADH (0.25 mM), NaN_3_ (15 mM in _dd_H_2_O, Sigma), oxaloacetate (0.47 mM and 0.14 mM), paraffin (Sigma), Permount (Fisher, SO-P-15), Pyruvate-Na (25 mM), rabbit anti-mouse CACNA1C antibody (Abcam, ab58552), rabbit anti-mouse SERCA2 ATPase (Abcam ab91032), rabbit anti-mouse NCX1 antibody (Cell Signaling Technology #79350), secondary anti-rabbit antibody (B700, Bio-Rad Starbright), secondary anti-mouse antibody (B700, Bio-Rad Starbright), secondary anti-rabbit antibody (Licor IRDye 800CW), Schiff’s reagent (1.2 mM basic fuchsin; 0.1 M HCl; 2.1 mM sodium metabisulfite), SOD assay kit (19160, Sigma), TGX stain-free fast cast acrylamide kit 12% (Bio Rad), triphenylphosphine (Sigma), UltraClear (J.T.Baker, Phillipsburg, NJ, USA), 2,7-dichlorofluorescein (CAS 76-54-0, Sigma), and 2,7-dichlorofluorescin diacetate (0.55 μM in KF-Buffer, CAS 4091-99-0, Sigma).

### Animals

Nine untrained and ten trained 3-month-old mice (males, C57Bl/6NCrl) weighing 29.2±0.7 g and 29.6±0.3 g (AVG±SEM) at the baseline respectively were used in the heart function measurements. The mice were maintained in standard laboratory conditions (24 ± 0.5 °C, 39 ± 7.6% air humidity) with 12-h light and dark cycle with ad libitum access to bottled tap water and pellet feed (SDS special diets services, CRM Expanded, UK). All the mice were housed individually in plastic cages (42×26.5×18.5 cm) with the cage bedding changed weekly (GLP aspen bedding, TAPVEI®, Estonia). The mice were handled in accordance with the institutional animal-care policies of the University of Turku. The study protocol was approved by the Animal Experiment Board Finland and the Ethical Committee of the University of Turku. The experiments were conducted with the following animal care license: KEK/2008-0206.

### Exercise protocol

The mice were raised in the central animal laboratory at the University of Turku, Finland. Upon arrival to the Department of Biology, the mice were acclimatized for 1 day after which they were weighed and separated randomly into two groups and given either a dummy wheel (does not spin, diameter 17.5 cm) (untrained group) or a low-profile wireless running wheel (diameter 15.5 cm, ENV-044, Med Associates Inc., San Diego, CA, USA) (trained group). An acclimatization time of 1 day was considered sufficient as the mice were in transit for less than half an hour and we had previously tested how long mice would take to start running in their new environment. For the trained mice, the wheel spins were recorded using wireless running wheel USB interface Hub (DIG-804, Med Associates Inc., VT, USA) and wheel manager software (SOF-860, Med Associates Inc., VT, USA). In the beginning, mice were weighed 2–3 times (start weight) and encouraged to start using the wheels by lifting them onto the wheel. Exercising mice were permitted to run over a period of 4 weeks.

### Heart function measurements

**Fig. 1:**
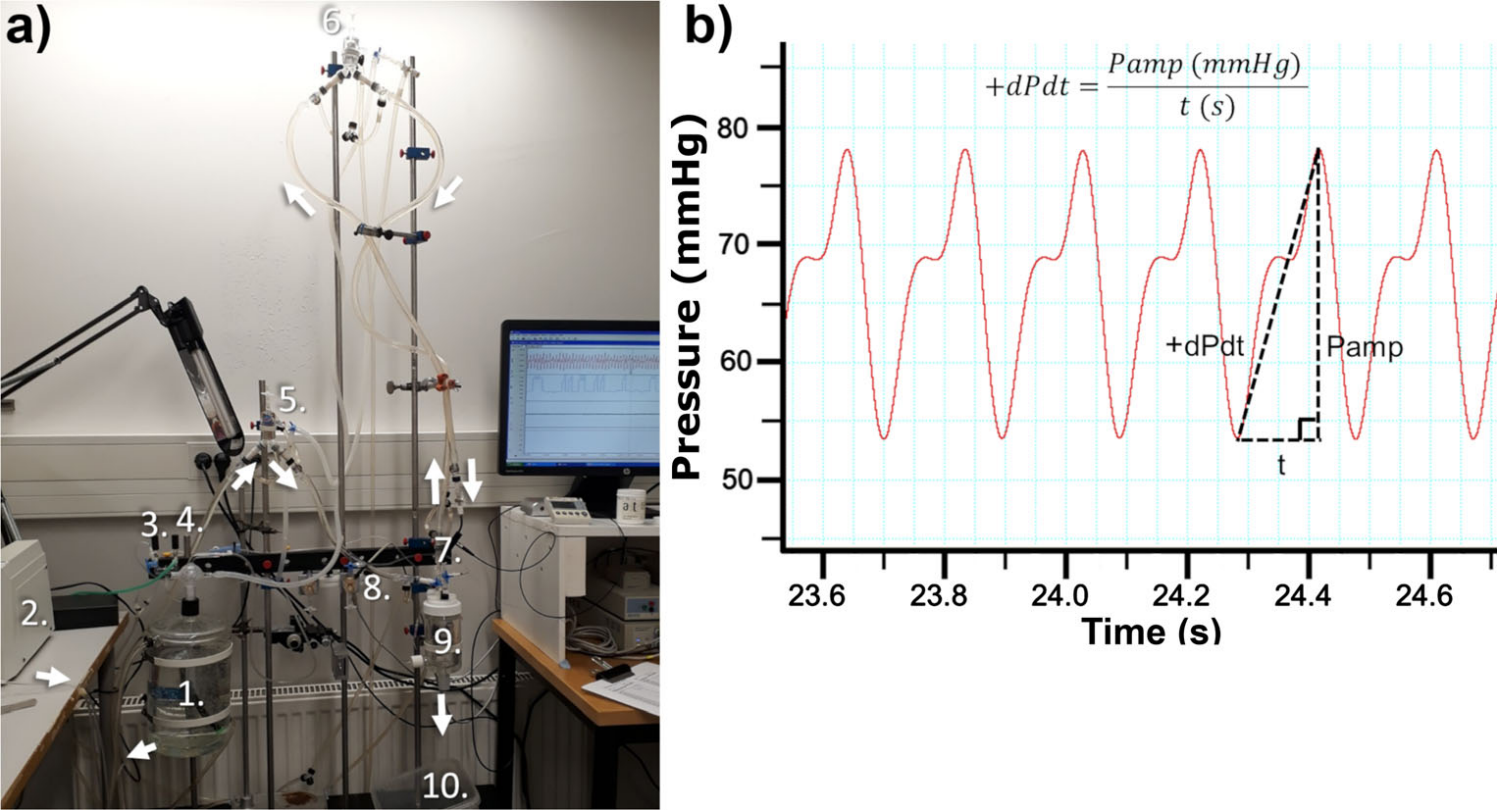
The Langendorff set-up (**a**) and the aortic pressure recording (**b**). Langendorff set-up consisted of perfusate reserve (1), peristaltic pump followed by a filter (2), oxygenation valve (3), nitrogen valve (4), bubble locks (5,6), oxygen sensor (7), aortic pressure sensor (8), chamber with temperature sensor and cannula (9) as well as waste collector (10) and warm water pump not shown in image. The white arrows indicate the direction of the perfusate flow. The heart rate, pressure amplitude, and rate of pressure generation and decrease (dP dt^-1^) were determined from the pressure recording

Mice hearts were removed during anesthesia with i.p. chloral hydrate (500 mg kg^-1^ of animal, JT. Baker®, Deventer, Holland) after i.v. heparin injection (100 μl, 3000 U in 0.9% NaCl, LEO Pharma Oy, Finland) for the prevention of blood clotting. After removal, the hearts were submerged in cold Krebs-Henseleit solution (NaCl 119 mM, NaHCO_3_ 25 mM, Glucose 11.1 mM, KCl 4.7 mM, KH_2_PO4 1.2 mM, MgSO_4_•7H_2_O 1.2 mM, CaCl_2_•2H_2_O 1.8 mM, oxygenated with 95% O_2_ 5% CO_2_). While submerged, the aortas were cannulated and then attached to the Langendorff heart perfusion apparatus (Fig. 1a) during constant perfusate flow. The hearts were subjected to non-recirculating retrograde perfusion with Krebs-Henseleit solution in constant pressure (70±3.5 mmHg) using a peristaltic pump (Masterflex® 77521-47, Cole-Parmer Instrument Company, Barrington, IL, USA). The circulating perfusate was kept warm with a heated circulating water bath (Digital One 070051A, Radnoti, Newington, NH, USA). The temperatures of the hearts were monitored using an implantable thermocouple probe (IT-18, MLT1401 T-type, and T-type Pod ML312, ADInstruments, Australia) and kept between 38±0.8 °C throughout the measurement. The perfusate was oxygenated with carbonated oxygen while the hearts were left to recover for 15–30 min. After this, carbonated nitrogen (95% N_2_ / 5% CO_2_) was used to gradually lower the oxygen content of the perfusate. The oxygen level was monitored using a fiber-optic oxygen meter (FireStingO_2_ PyroScience, Aachen, Germany) connected to a flow-through oxygen sensor cell (OXFTC, PyroScience, Aachen, Germany). A pressure sensor (SP 488 Memscap AS, Skoppum, Norway) located above the cannula was used to measure the perfusion pressure and changes in afterload pressure generated by the heart. The pressure sensor was connected to its preamplifier (Bridge Amp ML221, ADInstruments, Australia). The preamplifier was connected to PowerLab 8/30 (ADInstruments, Bella Vista, NSW, Australia) which was connected to a desktop PC. Recording of heart function was done continuously using Chart™ (v.5.5.1) until the oxygen content of the perfusate reached 5 mg l^-1^. Data points were set for every 1 mg l^-1^ decrease in oxygen level.

### Acquisition of heart function data and body composition data

The body mass indexes (BMI) of the animals were determined using a formula weight (kg) length^-2^ (m). The length of the animals was determined by measuring from the tip of the snout to the base of the tail. The relative heart weights were determined in two ways: by dividing heart weight by body weight (RHW, mg g^-1^) or by length (RHWL, mg mm^-1^). Exclusion criteria were set for the resting state of the isolated heart function to avoid obtaining data from injured hearts. The hearts had to reach sinus rhythm with no prolonged arrhythmias ( > 15 s) or visible blood clotting in coronaries [23]. Also, the hearts had to reach the minimal heart rate of 300 beats per minute (bpm) in oxygen levels ≥ 20 mg l^-1^ and minimal afterload pressure amplitude of 4 mmHg [23]. In lower oxygen levels during resting state, the following exclusion criteria were applied: 280 bpm and 4 mmHg in 19 mg L^-1^ of oxygen and 260 bpm and 4 mmHg in 18 mg l^-1^ of oxygen. To avoid bias in exclusion criteria, all exclusion criteria were applied before any analysis of data was done. From the heart function measurements, heart temperature, mean perfusion pressure, heart rate, afterload pressure amplitude (Pamp), and rate of pressure development in systole (dP dt^-1^) and rate of pressure decrease (-dP dt^-1^) in diastole were calculated for each oxygen level (Fig. 1b). The rate of pressure development or decrease in pressure was calculated in systole and diastole, respectively using the formula P t^-1^ (mmHg s^-1^). The oxygen level at which arrhythmia occurrence increased (arrhythmia breakpoint) was assessed visually and by comparing the recorded continuous arrhythmia durations and number of occurrences between consecutive oxygen levels. Disruptions in pressure development were used as indicators of arrhythmia. The oxygen level at which the heart rate began to decrease (heart rate breakpoint) was calculated using the breakpoint analysis function in SigmaPlot 14.0 (Systat Software, Inc. San Jos, CA, USA).

### Calcium channel analysis

After the Langendorff measurements, hearts were dried, weighed, and then cut longitudinally into the right and left halves (ventricle and atrium), and the right half was immediately flash-frozen in liquid nitrogen and stored at −80 °C. The frozen right half of the heart, containing partially both left and right ventricle as well as the right atrium, was crushed and the pieces were weighed and homogenized in 1:6 mg μl^-1^ of homogenization solution (62.5 mM Tris-HCl, 1μg ml^-1^ leupeptin, 1μg ml^-1^ pepstatin and 1 mM PMSF, pH 6.8) using two steel beads and TissueLyser (Qiagen, Cat. 85220, Germany) 2 × 1 min s^-1^. Tissue lysate was centrifuged at +4°C 5100g for 5 min and the supernatant was collected and aliquoted for protein measurement and western blot. Aliquots for protein measurement were flash-frozen and stored at −80 °C while the aliquots for western blot were diluted 1:1 in Laemmli solution (625 mM Tris pH6.8, 20% glycerol, 2% SDS, 0.025% bromophenol blue, β-mercaptoethanol 5%). Thereafter, the proteins were denatured at 70 °C for 7 min and then stored at −80°C. Protein concentrations were determined using bicinchoninic acid, BCA assay (Pierce®, Thermo scientific, USA). Using TGX stain-free fast cast acrylamide kit 12% (Bio-Rad) 10, 20, 5, and 20 μg of protein was separated for the antibody detection of the following proteins CACNA1C, RyR-2, SERCA2, and NCX1, respectively. Gels were imagined with a ChemiDoc™MP imaging system with Imagelab™Touch Software (BIORAD) for the calculation of total protein amount. Then the proteins were transferred to Amersham™ Protran™ nitrocellulose blotting membrane (0.45 μm, GE Healthcare Life Science, Germany). The membranes were blocked for one hour in TBS with 5% fat-free milk (5% BSA for NCX1) and stained overnight at +4°C in the primary antibody dilution of 1:1000 (1:2000 for SERCA) in the 5% fat-free milk or BSA TBS+0.1% Tween-20. Rabbit IgG antibody was used to detect CACNA1C, SERCA2 ATPase, and NCX1 (Abcam ab58552, Abcam ab91032, Cell Signaling Technology #79350) while mouse IgG antibody was used to detect RyR-2 (Sigma-Aldrich R128). The membrane was incubated with the secondary antibody 1 h at room temperature with the antibody being diluted 1:5000, using Bio-Rad Starbright anti-rabbit or anti-mouse B700 antibody or Licor anti-rabbit IRDye 800CW antibody. Membranes were all imaged using the ChemiDoc™MP imaging system (Bio-Rad).

Image lab 6.0 (Bio-Rad) was used to analyze the western blot band intensities from the gels and the membranes. Gel band intensities were used to calculate the overall protein content per lane for all the tested channels except for RyR-2, for which this could not be done accurately due to the long SDS-PAGE running time that was required to resolve the RyR-2 bands which resulted in the loss of a majority of the protein bands. The intensity of the ion channel band was divided by the overall protein content of the lane to minimize the effects of pipetting inaccuracies. Loading controls were used to calculate the relative values between gels to correct the final band intensities of all the calcium channels tested in case of possible variation between gels. Additionally, ratios between the relative amounts of calcium channels were calculated for CACNA1C and RYR-2 as well as for NCX1 and SERCA2.

### Enzymatic activity and oxidative stress measurements

For enzymatic activity measurements, the heart tissue was homogenized in 1:6 mg μl^-1^ of 100 mM K-phosphate buffer containing 150 mM KCl solution with pH7.4 (KF-buffer) or in methanol (500 μl). The homogenates in KF-buffer were diluted further in 50 mM Tris pH 8 for citrate synthase (CS) activity measurement and in 50 mM Tris pH 7.4 for lactate dehydrogenase (LDH) measurement. The undiluted homogenates in KF-buffer were centrifuged at +4°C, 10,000g for 15 min while methanol lysates were centrifuged at +4°C, 5100g for 10 min. Following centrifugation, the methanol-free supernatant was aliquoted into separate tubes for the measurement of protein concentration, superoxide dismutase (SOD), reactive oxygen species (ROS), and catalase (CAT) while the supernatant containing methanol was aliquoted solely for the lipid peroxidation (LPX) measurement. All the samples were flash-frozen in liquid nitrogen and stored at −80 °C until analyses. The CS activity was measured using a protocol described in Attila et al. (2013) with the use of the following substrate concentrations: DTNB 0.17 mM, oxaloacetate 0.47 mM and 0.14 mM [3]. The background signal was determined by measuring the signal without the addition of oxaloacetate. The LDH activity was also measured using a protocol described in Anttila et al. (2013) with the following final substrate concentrations: NADH 0.25 mM and 25 mM pyruvate-Na [3]. The background signal was determined by measuring absorbance without the addition of pyruvate-Na. Both CS-activity and LDH-activity measurements were done by measuring color formation 30 times within 2.3 minutes at 37 °C at a wavelength of 412 nm for CS and 340 nm for LDH. After measurements, the protein concentrations were determined for each sample using the BCA assay.

The SOD activity was measured by determining SOD inhibition percentage using the SOD assay kit (19160, Sigma) at a protein concentration of 0.3 mg ml^-1^. The kit measures SOD inhibition which is directly correlated with the sample SOD activity. The kit protocol was adjusted to a 384-well plate as was done by Stauffer et al. (2018) and the absorbance was measured at 450 nm twice, first for background signal detection before the addition of enzyme and then for signal detection after enzyme addition and incubation at 37 °C for 20 min [22].

The ROS level measurement method was modified from the method described in Socci et al. (1999) and in Lilley et al. (2014) using 1 mg ml^-1^ protein dilution. 2,7-Dichlorofluorescein (CAS 76-54-0, Sigma) was used as a standard and 2,7-dichlorofluorescin diacetate (CAS 4091-99-0, Sigma) was used for the reactions, all of which were diluted in KF-buffer [15, 21]. The fluorescence was measured with an excitation wavelength of 485 nm and an emission wavelength of 535 nm. Measurement was done by pipetting 50 μl of standard and samples in quadruplicate, with the fourth well of the samples being used for background autofluorescence detection (defined as blank). Five microliters of dilution buffer was added to all standard wells and into the blank well, while 5 μl of 2,7-dichlorofluorescin diacetate (0.55 μM) was added to the three first sample wells. The samples were incubated for 10 min and then measured.

The CAT activity was measured according to the protocol that is described in Vuori and Kanerva (2018) at a protein concentration of 0.6 mg ml^-1^ [24]. Catalase reaction was stopped with H_2_O_2_ (200 mM) using NaN_3_ (15 mM in _dd_H_2_O) and then the remaining H_2_O_2_ was detected with colorimetric reaction at a wavelength of 544 nm by using H_2_O_2_ as a standard.

The lipid peroxidation was estimated by measurement of lipid hydroperoxides (LHPs) using a modified method from Raja-Aho et al. (2012) [20]. The absorbance was measured at 570 nm. To remove non-lipid peroxidation from the results, triphenylphosphine was added to sample aliquots before the absorbance measurement. A cumene hydroperoxide in methanol was used as a standard and all samples were measured after 1-h reaction incubation in dark at room temperature.

All the absorbance measurements were done using transparent 96- or 384-well plates and the fluorescence detection was done using dark opaque 384-well plates. Measurements were done using a multilabel plate reader (Perkin Elmer, EnSprire 2300, or Envision 2103).

### Histology

The left hearts were fixed in 10 % formalin and stored in 70% ethanol until sectioning. The hearts were cut in half transversally and dehydrated in increasing alcohol series (94% Ethanol, 3×100% ethanol) for 1–2 h depending on the tissue size and by incubation in UltraClear (J.T. Baker, Phillipsburg, NJ, USA, 2×1–2h) and then infiltrated with paraffin for 1–2 h and again in fresh paraffin overnight (Aldrich 76242). The samples were cut into 5-μm sections which were deparaffinized in UltraClear and rehydrated in decreasing alcohol series for capillary staining. The capillary staining was done using periodic acid-Schiff staining protocol according to Andersen (1975) using 35-min Schiff’s reagent staining (1.2 mM basic fuchsin; 0.1 M HCl; 2.1 mM sodium metabisulfite) [2]. After staining the samples, sections were dehydrated in increasing alcohol series and in UltraClear and then coverslipped using Permount histological mounting medium (Fisher, SO-P-15). All the imaging was done with Nikon Eclipse microscope using Nikon pE-300ultra camera and NIS-Elements AR-software. Histological images were analyzed using Image J 1.52a. The capillary density was counted from a minimum area of 20,000 μm^2^ of tissue. From these areas, the cell number was also determined which was used to count the average cell size and the capillary to cell ratio.

## Statistical analysis

All the data are shown as mean ± standard error and all the statistical tests were performed using SigmaPlot 14.0. The data were tested for normality and variance using Shapiro-Wilk and Brown-Forsythe, respectively. Heart function data were compared between the groups using repeated measures two-way ANOVA, with oxygen level and test group as factors. The heart function data were not normally distributed, and logarithmic transformation did not improve the normality. If the variance was improved via logarithmic transformation, the transformed data were utilized for the statistical tests; otherwise, original data were utilized. When ANOVA was statistically significant for interaction, the Holm-Sidak post hoc test was performed. The Student *t*-test was used to compare the groups for the arrhythmia and heart rate breakpoints, the relative amounts and the ratio of calcium channels, weight gain, BMIs, heart weights, RHWs and RHWLs, and the activity of CS and LDH. In cases when the variances were not equal, Welch’s t-test was used to test the differences between groups. If the data were not normally distributed, the differences between groups were tested using the Mann-Whitney rank-sum test. The outliers for non-repeated measurements were removed if their values differed from the group mean twice by the standard deviation or more. Correlation analysis was done within groups on several of the measured variables using linear regression analysis. Since CACNA1C and RyR-2 both contribute to the speed of contraction and the power of contraction, correlations were determined for systolic dP dt^-1^ and Pamp within oxygen level of 18 mg l^-1^ and between the amount of CACNA1C and RyR-2 as well as the ratio between these two channels. SERCA2 and NCX1 contribute to the relaxation of the heart, so correlations were also determined between the rate of pressure decrease in diastole within oxygen level of 18 mg l^-1^ and the amount of SERCA2 and NCX1 as well as the ratio between these two channels. The mean perfusion pressure was also tested for correlation with the systolic pressure production to ensure that small amounts of variability did not alter heart function. To ensure the cannulation time did not affect the results linear regression analysis was also performed between the cannulation time, the systolic pressure production, and Pamp. The heart function data from an oxygen level of 18 mg l^-1^ were used for all the correlation tests because this was the highest oxygen level with the greatest n-number. The starting oxygen level for each heart varied depending on the heart’s perfusion flow speed.

## Results

At the end of the exercise period, the animals had run a total of 84.8 ± 8.0 km with an average of 3.2 ± 0.3 km day^-1^ (AVG ± SEM). The test groups did not differ in most of the measured body composition variables (Table 1). However, the trained animals gained significantly less weight than the untrained animals (Table 1).

**Table 1:** Body mass and heart mass of trained and untrained mice (average ± SE)

	Untrained (*n*= 9)	Trained (*n* = 10)	Test value	*p* value
BMI (kg m^-2^)	3.6 ± 0.1	3.5 ± 0.04	Wt = 0.531	0.606
Weight gain (g)	1.8 ± 0.4	-0.2 ± 0.3	t = 3.655	**0.0021**
Heart weight (mg)	237.5 ± 9.6	254.2 ± 11.6	t = -1.032	0.316
RHW (mg g^-1^)	7.7 ± 0.2	8.5 ± 0.3	t = -1.972	0.065
RHWL (mg mm ^-1^)	2.6 ± 0.1	2.8 ± 0.1	t = -1.356	0.193

*BMI* body mass index, *RHW* relative heart weight, *RHWL* relative heart weight to body length. The statistical significance was tested with t-tests (t) or with Welch’s t-test (Wt) if data did not have equal variance. One outlier was removed from the trained group weight gain analysis. Statistically significant result is shown in bold

The heart temperature did not differ between the test groups or throughout the measurement between different oxygen levels, but the mean perfusion pressure did differ (*F* = 5.677, *p* = 0.029). The perfusion pressure was greater in the exercising test group than in the untrained group throughout the measurement (AVG ± SEM: 71.9 ± 1.0 mmHg; 68.4 ± 1.1 mmHg). Additionally, the perfusion pressure was not affected by the oxygen level and there was no interaction between the oxygen level and test group. However, there was no significant correlation between the perfusion pressure and the systolic pressure development (online resource 1) nor between cannulation time and either the systolic pressure development or the pressure amplitude at an oxygen level of 18 mg l^-1^.

**Fig. 2:**
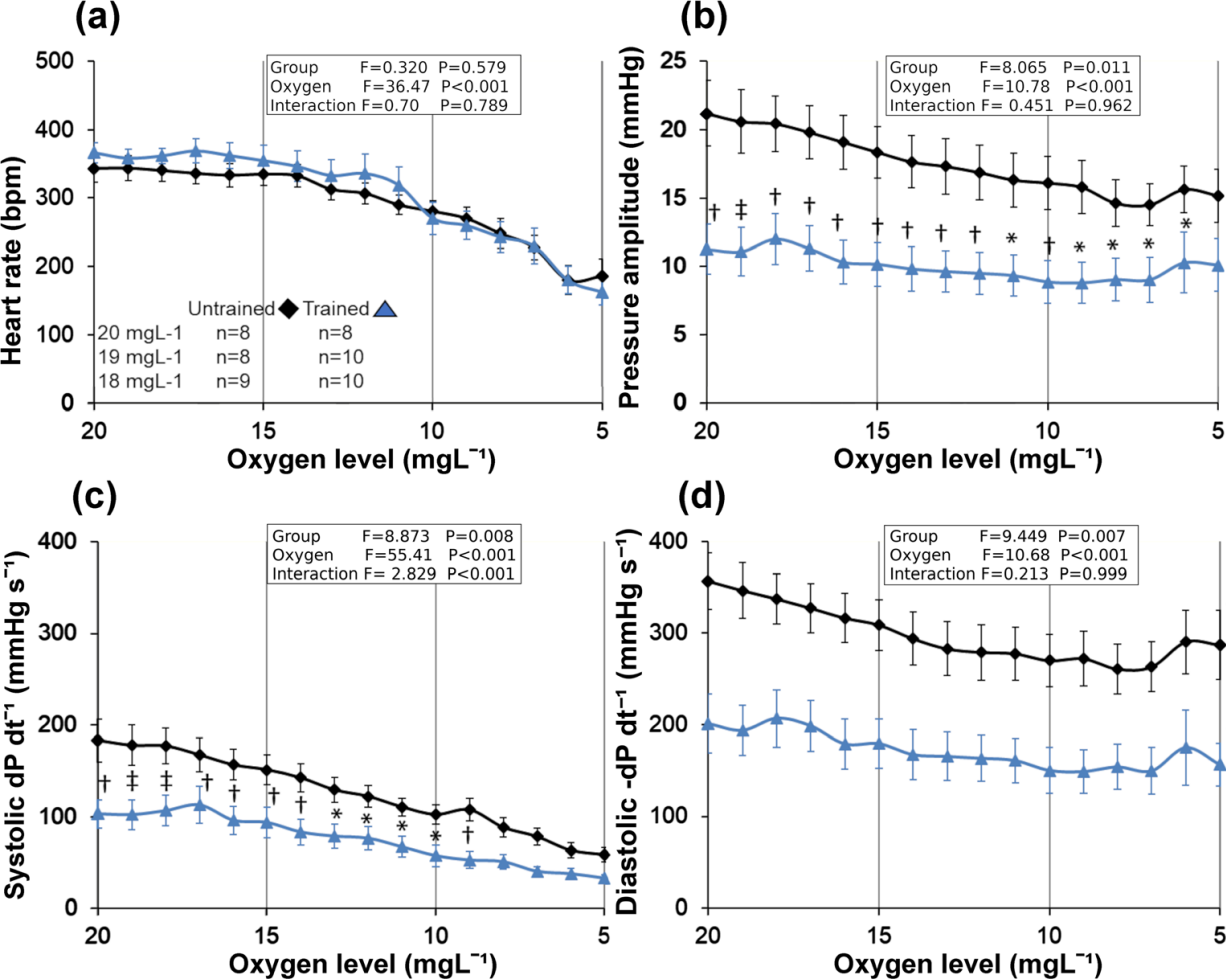
Heart rate is shown in beats per minute (**a**), afterload pressure amplitude (**b**), and rate of pressure development (dP dt^-1^) in systole (**c**) and decrease (-dP dt^-1^) in diastole (**d**) in untrained and trained mice in different oxygen levels ex vivo. The starting oxygen level for each heart varied depending on the heart’s perfusion flow speed, therefore n-number is shown for the highest oxygen levels (**a**). The sample size remains *n*=9 in untrained and at *n*=10 in the trained group at oxygen levels below 18 mg l^-1^. Holm-Sidak post hoc ‡*p*≤0.001, †*p*≤0.01, *p<0.05. The statistical test values of ANOVA tests are shown as indexes

The heart rate of the animals slowed down significantly (*p* < 0.001) as the oxygen levels decreased (Fig. 2a). There were no differences in heart rate between the groups or interaction between oxygen level and test group (Fig. 2a). Pressure amplitude, on the other hand, was lower in the trained group (*p* = 0.011) (Fig. 2b) but there was no interaction between the main factors. As with the heart rate, the oxygen level significantly affected the pressure amplitude (*p* < 0.001) causing it to decrease as the oxygen level decreased (Fig. 2b). The systolic rate of pressure development also decreased as the oxygen level dropped (*p* < 0.001) (Fig. 2c). Furthermore, there was an interaction between the factors in the systolic rate of pressure development (*p* < 0.001, Fig. 2c). The rate of pressure development in systole differed between the trained and untrained test groups (*p* = 0.008, Fig. 2c). The rate of pressure development in systole was greater in the untrained group, but it declined more steeply with the decreasing oxygen levels as compared to that in the trained group (Fig. 2c). Indeed, the post hoc test revealed that the rate of systolic pressure development in the untrained test group remained significantly faster only in oxygen levels ≥8 mg l^-1^ as compared to the trained group (Fig. 2c). The rate of pressure declines during diastole also decreased along with decreasing oxygen levels (*p* < 0.001) (Fig. 2d). Although there was no interaction between the factors, the rate of pressure decline was slower in the trained group as compared to that in the untrained group (*p* = 0.007) (Fig. 2d).

**Fig. 3:**
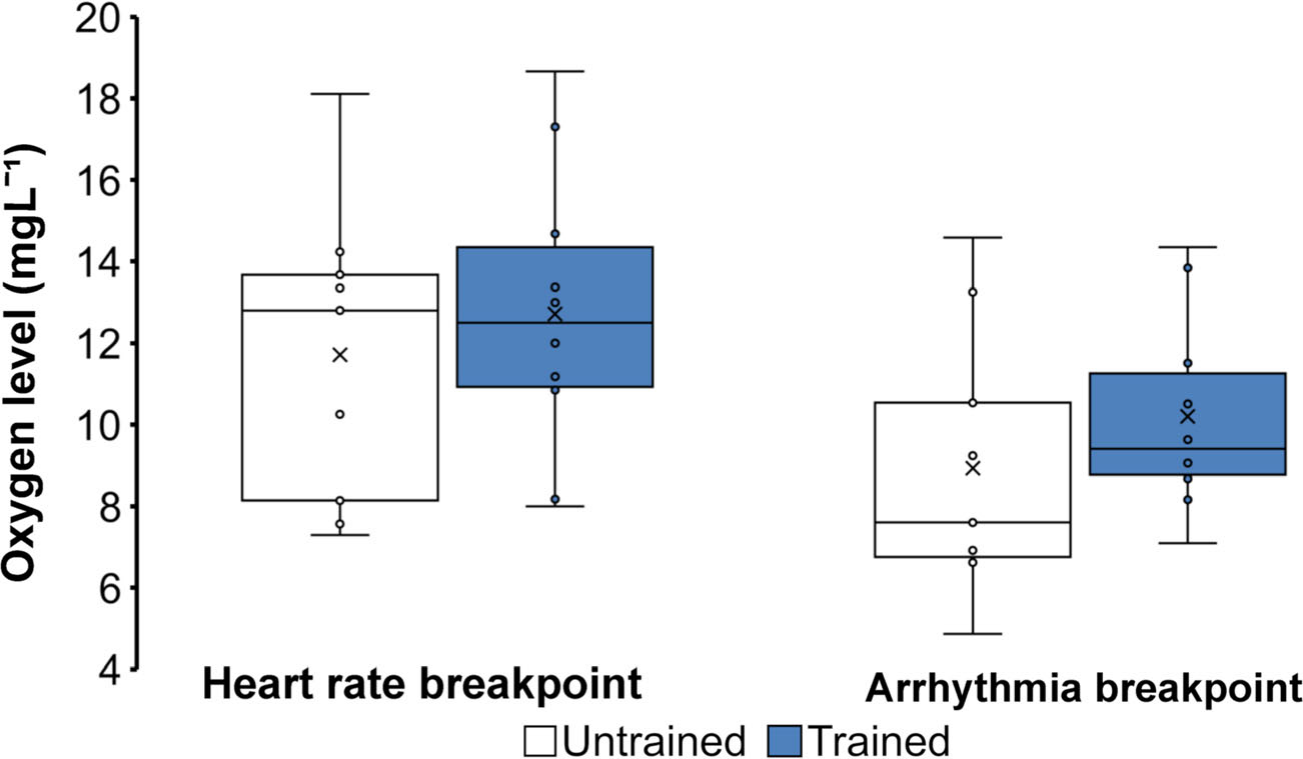
The oxygen levels at which the heart rate started to reduce significantly (heart rate breakpoint) and where significant arrhythmias were observed (arrhythmia breakpoint) in untrained and trained mice. Disruptions in pressure generation were used as an indicator of arrhythmia occurrence. Whiskers indicate maximal and minimal values, without outliers, while the box upper line and lower line indicate upper and lower inclusive quartiles respectively with the middle line indicating the group median and x the group mean

**Fig. 4:**
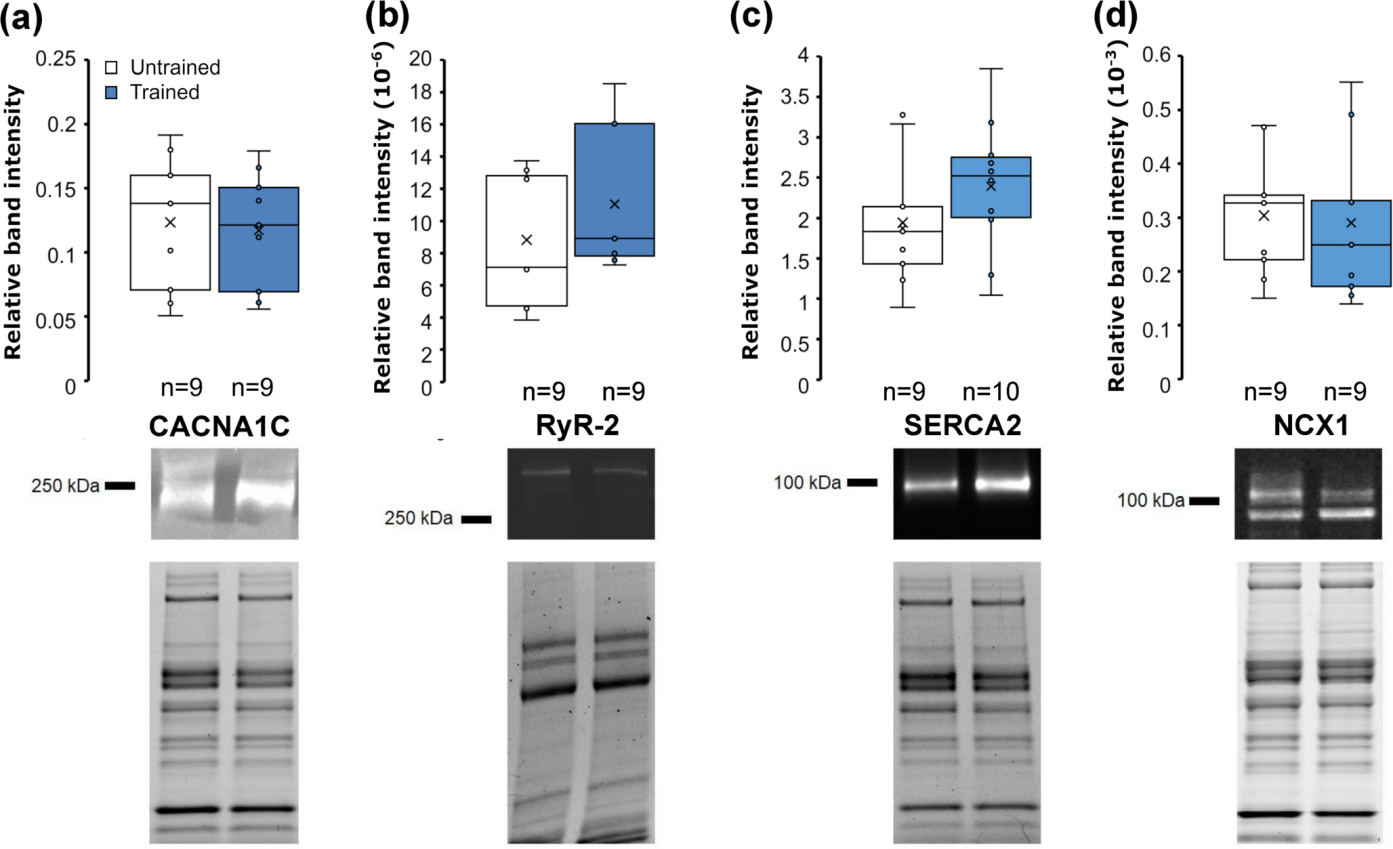
The level of cardiac calcium channel proteins in the untrained and trained mice. The band intensities are shown relative to the overall protein amount for CACNA1C (**a**), RyR-2 (**b**), SERCA2 (**c**), and NCX1 (110kDa) (**d**). Whiskers indicate maximal and minimal values, without outliers, while the box upper and lower line indicate upper and lower inclusive quartiles respectively with the middle line indicating the group median and × the group mean. The images represent the bands of respective proteins and the overall protein band intensities of samples analyzed

There were no significant differences between trained and untrained animals in terms of the oxygen levels at which they started to experience arrhythmias, or their heart rate began to decline (Fig. 3). The CS activities were 0.45±0.02 μmol × mg^-1^ min^-1^ and 0.49±0.04 μmol × mg^-1^ min^-1^ for the untrained and trained group respectively, and there was no statistically significant difference between these values. The LDH activities were 0.06±0.004 μmol × mg^-1^ min^-1^ and 0.07±0.006 μmol × mg^-1^ min^-1^ for the untrained and trained groups respectively, and likewise, there was no statistically significant difference between these values. Additionally, none of the oxidative stress markers nor the histological parameters differed significantly between the groups (data not shown), although the SOD inhibition percentage had a trend of being lower in the trained group compared to the untrained group (*p*=0.0585, 68.16±1.6%, and 72.02±1.8%, respectively). Furthermore, the untrained and trained group did not differ in terms of their levels of CACNA1C, RyR-2, SERCA2, or NCX1 (Fig. 4). Neither the ratio of CACNA1C/RyR-2 nor NCX/SERCA2 differed significantly between the groups, although the ratio of CACNA1C/RyR-2 decreased in the trained group as compared to the untrained group (online resource 2).

**Fig. 5:**
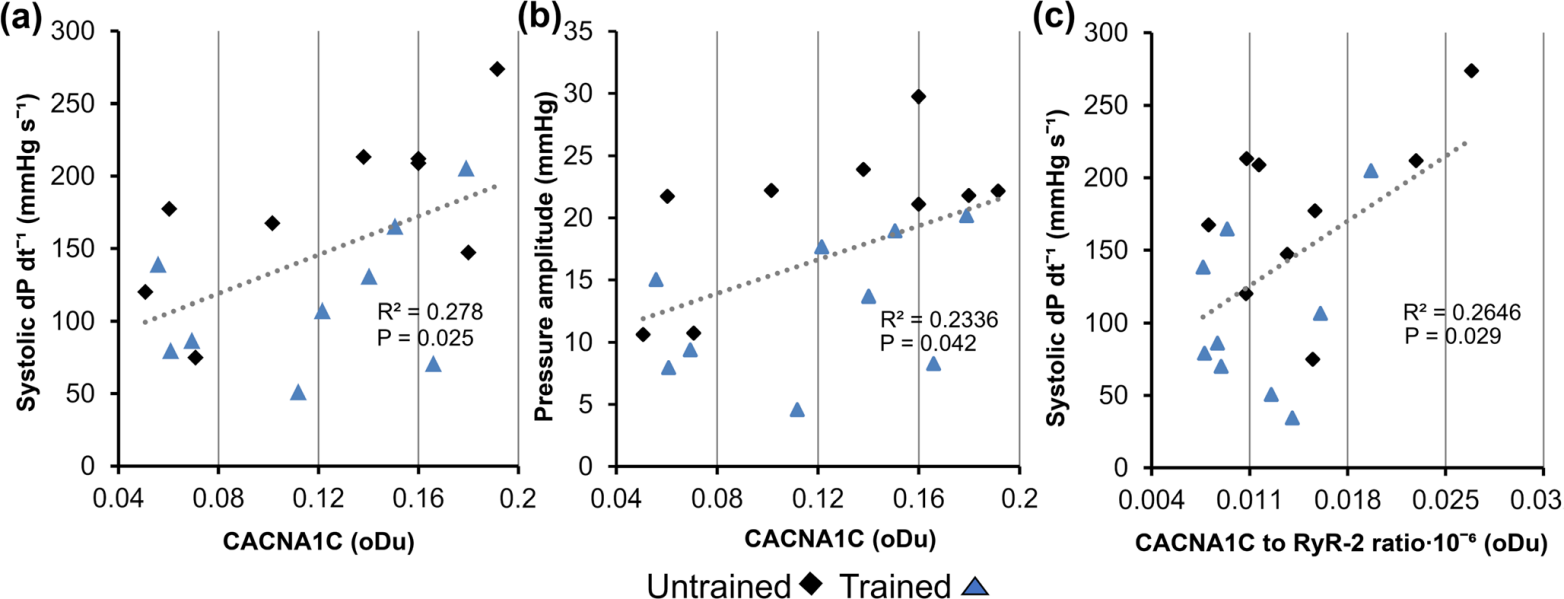
The correlation of CACNA1C protein level with the systolic pressure production (**a**) and pressure amplitude (**b**) as well as the correlation between CACNA1C to RyR-2 ratio and systolic pressure production in the oxygen level of 18 mg l^-1^. Linear regression *P* and *R*^2^-values are shown as indexes. *n* = 9 in both groups

The rate of pressure development in systole correlated positively with the amount of CACNA1C (*R*^2^ = 0.278, *p* = 0.025, Fig. 5a) and there was also a significant positive correlation between CACNA1C protein level and the afterload pressure amplitude (*R*^2^ = 0.234, *p* = 0.042, Fig. 5b). Further correlation tests also revealed that the CACNA1C to RyR-2 ratio had a significant positive correlation with the rate of pressure generation (*R*^2^ = 0.265, *p* = 0.029, Fig. 5c), but not with afterload pressure amplitude. There were no significant correlations between RyR-2 and pressure amplitude nor RyR-2 and rate of pressure generation. Likewise, there was no correlation between SERCA2 or NCX1 and the rate of pressure decrease.

## Discussion

We demonstrated that a 4-week period of voluntary running wheel training is insufficient to improve intrinsic murine cardiac hypoxia tolerance. However, interestingly, the trained animals exhibited decreased amplitude and rate of cardiac pressure production and relaxation in normoxia and hypoxia when compared to untrained animals with a similar heart rate. Previously, exercise-induced bradycardia has been shown to persist ex vivo after a 4-week swimming training regimen in mice [7]. The training protocol in our study did not induce bradycardia, which may be due to insufficient intensity of exercise due to voluntary on-and-off wheel running. Instead, it seemed that the heart rate was not altered in the trained animals at any of the oxygen levels when compared to the untrained group. However, in contrast to the previously mentioned study and in accordance with our results, a study by Lakin et al. (2018) showed that both forced swimming (6 weeks) and voluntary running exercise (6 weeks) induced bradycardia in vivo through parasympathetic nervous activity and that the bradycardia did not persist *ex vivo* or after autonomic blockade [13]. Therefore, it is possible that the intensity of exercise in our study was not sufficient to induce intrinsic changes in the heart, but that there may have been alterations induced by the autonomous nervous system. Additionally, the results from the study by Lakin et al. indicate that the mode of exercise, forced versus voluntary, is unlikely to explain the lack of alterations in our study [13].

Heart rates higher than 400 beats per minute have been shown to lower left ventricular developed pressure amplitude, cardiac power, and the rate of pressure development (dP dt^-1^) in the ex vivo mouse heart [14]. However, the average heart rates within this study did not differ between the groups and they were also under 400 beats per minute in all oxygen levels tested; therefore, heart rate cannot fully explain the decreased pressure production of the trained hearts. Although pressure changes measured in afterload pressure above the aorta are not directly translatable to the intraventricular developed pressure measured in a non-recirculating retrograde perfused heart, they have been shown to be indicative of the intraventricular pressure changes and, thus, the force and power at which the heart contracts [16]. An intra-ventricular balloon was not used in this study to allow faster measurement and easier preparation of the heart, thus allowing more animals to be measured on a single day. Our results indicate that the trained hearts in this study had slower pressure generation capacity as compared to untrained mice, and therefore, the force of the contraction remained lower as well. Interestingly, the pressure decrease was also slower in the trained hearts in our study, indicating slower relaxation. Our results contradict in vitro heart studies in which training has been shown to improve cardiac fiber contractility [28, 29]. Nevertheless, some studies have also found that the contractility of isolated cardiomyocytes is decreased in trained hearts despite training-induced ventricular hypertrophy [19]. These discrepancies could be due to fiber heterogeneity or variability between the measurement conditions such as external calcium levels and the pacing frequency used. Additionally, differing intensities and durations of exercise could explain the different results, although the decreased ventricular function has, to our knowledge, previously been found only after strenuous exercise as a part of so-called cardiac fatigue which is reversible [27]. However, it seems unlikely that the animals in the present study would voluntarily run enough to induce cardiac fatigue.

Hypoxia-induced cardiac hibernation could possibly also explain the decreased pressure production that we observed. Cardiac hibernation is characterized by the reversibly reduced contractile function of the heart and downregulated energy metabolism in response to ischemia and decreased coronary flow [10]. It has been postulated that this is an active cardiac adaptation to a lack of oxygen. Therefore, it could be theorized that the lower pressure production in the trained hearts of this study may indicate an improved capability of the trained hearts to adapt to hypoxic conditions and avoid resulting damage. However, the endpoint of hypoxia tolerance, the oxygen level at which arrhythmia occurrence increased, did not differ between the groups. Additionally, the pressure production was significantly lower in the trained hearts starting from the highest oxygen levels, indicating that the decrease in oxygen concentration did not induce this decrease in pressure generation. However, it is possible that the isolation process and resulting exposure to hypoxic conditions could have already induced cardiac hibernation, especially in the trained hearts. However, the CS and LDH activities were not significantly different in the trained versus untrained hearts which would suggest the trained hearts did not adjust their metabolism to better adapt to the hypoxic conditions than the untrained heart. Furthermore, the trained and untrained mouse hearts had similar levels of reactive oxygen species, antioxidative enzyme activities, and lipid peroxidation, suggesting the trained hearts were not able to significantly decrease their oxidative stress in response to hypoxia despite having lower pressure production. Additionally, the similar capillary density and unchanged cell size support our finding that hypoxia tolerance was not altered in response to this level of exercise.

A study by Fuller and Nutter (1981) showed significantly lower dP dt^-1^ of trained hearts when measured in situ, but in their study, the decreased function was attributable to the lower heart rate of trained animals [8]. The trained animals also have larger stroke volume; thus, the trained animals maintained similar cardiac output as compared to untrained ones [8]. The stroke work indexes indicated that in similar end-diastolic pressures in situ, the trained hearts and sedentary hearts had a similar cardiac performance at many different levels of preload [8]. In the present study, the preload conditions were the same for all the hearts, as the hearts were perfused in retrograde with no ventricle filling; therefore, the differing results reported in previous studies may be due to autonomic nervous regulation. It may also be that some functional alterations are not present in the heart during its resting state, and this could potentially even explain the lowered function of the trained hearts as they may have improved function during the non-resting state, although further studies are required to confirm this. Indeed, a study by Natali et al. (2001) found that a 6-week voluntary running-wheel exercise regimen does not significantly improve the contractility of isolated cardiac fibers when they are not under mechanical load, but under mechanical load, the trained heart cardiomyocytes produced greater force [17]. Our results also suggest that a trained heart might better sense loading conditions and therefore generate less force if it does not need to pump against heightened pressure.

Possible changes in ventricular volume were not looked at in this study, but in retrograde perfusion, no perfusate enters the heart which means that possible ventricular volume changes cannot explain the lower pressure production observed in trained animals. There is, however, a slight possibility that the somewhat larger hearts of trained animals could contribute to the rate of pressure generation (animals with bigger hearts often have a slower rate of contraction) but this does not explain the differences in the amplitude of pressure generation. For example, Kemi et al. (2005) found that 10-week treadmill exercise improved cardiomyocyte fractional shortening and caused cardiomyocyte hypertrophy in an exercise intensity-dependent manner, while also slowing the rate of cardiomyocyte contraction and relaxation [11]. It should also be noted that although the mean perfusion pressure was different between the groups, it seems unlikely that this would have affected pressure production in a significant way as there was no correlation between the rate of pressure production and the mean perfusion pressure. Additionally, measurement was done using constant pressure mode instead of constant flow mode which means perfusate is not forced into the coronaries to keep the flow rate constant. This allows the intrinsic coronary tone regulation within the heart to be preserved. This should secure coronary perfusion according to demand and this should further diminish possible effects of slight variations in mean perfusion pressure.

Since there was a significant correlation between the rate of pressure generation and the level of CACNA1C and its ratio to RYR-2, it is possible that even the small differences in the cardiac calcium channel levels and ratios may partially contribute to the changes in heart function. The small increase in the protein level of sarcoplasmic reticulum channels, SERCA2, and RyR-2 in trained animals could indicate that the trained hearts possibly have enhanced calcium current between the cytosol and sarcoplasmic reticulum. In agreement with our findings, training has been shown to increase the expression of SERCA2 and RyR in previous studies [7, 28, 29]. However, the calcium channel levels in the current study were not significantly altered so they cannot alone explain the alterations in heart function despite the significant correlations detected. It could be that the duration, intensity, or mode of exercise in this study was insufficient to significantly alter the expression of these calcium channels. Indeed, the previously mentioned studies utilized either a longer duration of exercise using rats, or the same duration but forced swimming exercise which was most likely of greater intensity. Nevertheless, even the small decrease in the ratio of CACNA1C to RyR-2 in trained hearts could further indicate that the trained hearts might rely more on the calcium of the sarcoplasmic reticulum to generate the calcium current for contraction. This means that the trained animals might rely less on the calcium current that CACNA1C generates, which was also the only channel tested that displayed a significant correlation with the rate of pressure generation and the overall generated pressure amplitude (Pamp). The smaller relative amount of CACNA1C in trained hearts could, therefore, partially explain the slower rate of pressure generation and the smaller developed pressure amplitude. Indeed, there was a significant positive correlation between the CACNA1C to RyR-2 ratio and the rate of pressure generation at the oxygen level of 18 mg ml^-1^. The correlation between the relative amount of CACNA1C and the rate of pressure generation is logical as CACNA1C initiates the calcium current into the cytosol and causes the RyR-2 to release calcium from the sarcoplasmic reticulum into the cytosol, which finally leads to contraction. Increased RyR-2 activity in the sinoatrial node has been shown to lead to decreased L-type calcium current in vitro, which is the calcium current via CACNA, through increased cytosolic levels of calcium which inhibit CACNA activity [18]. Therefore, one might speculate that the CACNA activity could have been affected in the trained hearts with higher RyR protein levels, which would further support the notion that trained hearts might rely more on intra-sarcolemmal calcium for contraction. However, the change in generated pressure amplitude, which should depend on contraction strength, cannot be explained by the changes in cardiac calcium channel protein levels. In particular, the increased amount of RyR-2 should lead to an increase of force of the contraction [9, 32] since RyR-2 is responsible for the calcium current which raises the cytosolic calcium concentration to a level that initiates contraction. Lastly, it seems paradoxical that the rate of pressure decrease was reduced in the trained hearts at diastole when there was a slight, although not significant, increase in SERCA2 protein levels. SERCA2 is involved in the removal of cytoplasmic calcium during diastole and should therefore improve the relaxation by allowing faster removal of calcium from the cytosol. Although not statistically significant, the increased level of SERCA2 could be a compensatory response to altered cytosolic calcium levels, which might explain why the rate of relaxation in trained hearts was not faster than in untrained hearts; however, it remains unclear as to why they were significantly slower instead.

It is also possible that the activity, as opposed to the level of these calcium channels, was altered. Further investigations are therefore warranted to reveal whether calcium channel phosphorylation can better explain the lowered pressure generation within trained hearts ex vivo. Unfortunately, this was not possible in the current study since all the hearts were measured for function until a very low oxygen level. At this point, they started to display disruptions in pressure development, which were indicative of severe arrhythmias. Such severe functional disruptions most likely alter the phosphorylation of the calcium channels themselves, and the activity of calcium channel inhibitors such as phospholamban. Future studies should, therefore, analyze the phosphorylation kinetics of channels without direct measurements of functional changes in severe hypoxia from the same hearts.

## Conclusions

The effects of voluntary wheel running have not been yet studied on the intrinsic hypoxia tolerance at multiple levels of hypoxia independent of reperfusion injury in the murine heart. This study shows that a 4-week voluntary running wheel exercise regimen is insufficient to improve intrinsic murine cardiac hypoxia tolerance at multiple levels of hypoxia and that trained mice exhibit lowered intrinsic frequency and amplitude of cardiac pressure production and slower pressure decrease in diastole when compared to untrained animals with similar heart rates. These effects cannot fully be explained by changes in the amount of cardiac calcium channels CACNA1C, RyR-2, SERCA2, or NCX1 even though the CACNA1C correlated positively with the rate of pressure generation and generated pressure amplitude. Similar results have not been shown before and they call for further investigation into the effects of exercise on intrinsic cardiac function studied ex vivo and reasons why the rate of the pressure generation and rate of pressure decrease is reduced in mice after 4 weeks of voluntary exercise training.

## Supplementary information

ESM 1 (PDF 166 kb)

## Abbreviations

AVG: average
BCA: bicinchoninic acid
BMI: body mass index
bpm: beat per minute
BSA: bovine serum albumin
CACNA1C: L-type Ca^2+^-channel
CAT: catalase
CS: citrate synthase
DTNB: 5,5′-dithiobis(2-nitrobenzoic acid)
dP dt^-1^: rate of pressure development (afterload pressure)
-dP dt^-1^: rate of pressure decline (afterload pressure)
i.p.: intraperitoneal
i.v.: intravenous
LDH: lactate dehydrogenase
LPX: lipid peroxidation
NADH: nicotinamide adenine dinucleotide
NaN_3_: sodium azide
NCX1: sodium-calcium exchanger
Pamp: pressure amplitude (afterload)
PMSF: phenylmethylsulfonyl fluoride
ROS: reactive oxygen species
RHW: relative heart weight
RHWL: relative heart weight to body lenght
RyR-2: ryanodine receptor
SDS: sodium dodecyl sulfate
SEM: standard error of mean
SOD: superoxide dismutase
SERCA2: Sarco(endo)plasmic reticulum Ca2+-ATPase
TBS: tris buffered saline
