# Supplementary material for: Short-term exercise affects cardiac function ex vivo partially via changes in calcium channel levels, without influencing hypoxia sensitivity
Source: J Physiol Biochem. 2021 Aug 27;77(4):639–51. doi: 10.1007/s13105-021-00830-z (PMC8605979; doi:10.1007/s13105-021-00830-z)
Supplement: Supplementary file 1 — (PDF 166 kb) [file 13105_2021_830_MOESM1_ESM.pdf]

Short-term exercise affects cardiac function ex vivo partially via changes in calcium channel levels, without influencing hypoxia sensitivity.

Journal of Physiology and Biochemistry

Tytti-Maria Uurasmaa[1*], Tomi Streng[1], Milla Alkio[1,2], Ilkka Heinonen[34#] & Katja Anttila[1#]

1 Department of Biology, University of Turku, 20014 Turku, Finland

2 Poznan University of Medical Sciences, Poznań, Poland

3 Turku PET Centre, University of Turku, and Turku University Hospital, 20014 Turku, Finland

4 Rydberg Laboratory of Applied Sciences, department of Environmental- and Biosciences, University of Halmstad, Halmstad, Sweden.

* Corresponding author: Tytti-Maria Uurasmaa, *Department of Biology, University of Turku, 20014 Turku, Finland*, Email: tmeuur@utu.fi

Equal last authorship

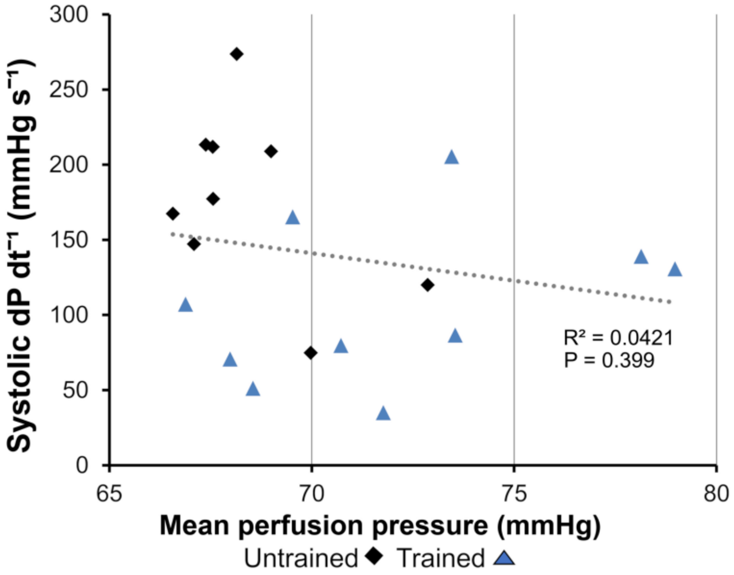

Online resource 1 Mean perfusion pressure correlation with the systolic rate of pressure production (dP dt^{-1}) within the oxygen level of 18 mg L^{-1}. Linear regression P and R^2-values are shown as index. Untrained group n = 9 and trained group n = 10

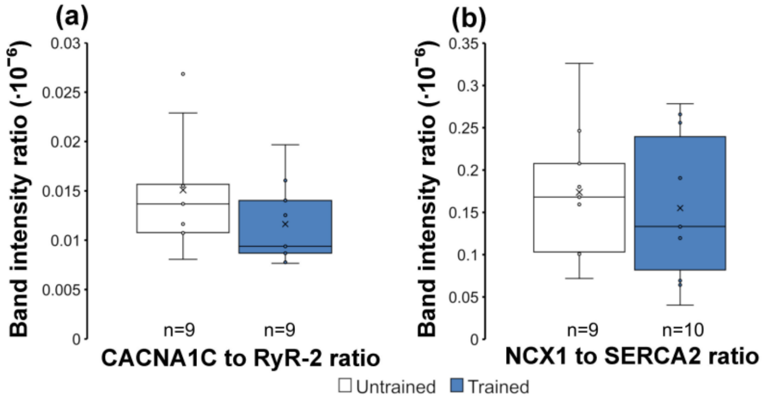

Online resource 2 The relative protein levels of cardiac calcium channels in the untrained and trained mice. CACNA1C to RyR-2 ratio (a) and NCX1 to SERCA2 ratio (b) are shown with whiskers indicating the maximal and minimal values, without outliers, while the box upper and lower line indicate upper and lower inclusive quartiles respectively with the middle line indicating the group median and x the group mean
